# Unveiling Neolithic Economic Behavior: A Novel Approach to Chert Procurement at Çukuriçi Höyük, Western Anatolia

**DOI:** 10.1007/s10816-024-09681-6

**Published:** 2024-12-24

**Authors:** Michael Brandl, Maria M. Martinez, Christoph Hauzenberger, Peter Filzmoser, Bogdana Milić, Barbara Horejs

**Affiliations:** 1 Austrian Archaeological Institute, Austrian Academy of Sciences, Dominikanerbastei 16, 1010 Vienna, Austria; 2 Amerind Foundation, Dragoon, AZ USA; 3 Department of Anthropology, The University of Texas at Austin, Austin, USA; 4 Department of Earth Sciences, University of Graz, Universitätsplatz 2, 8010 Graz, Austria; 5 Institute of Statistics and Mathematical Methods in Economics, TU Wien, Wiedner Hauptstrasse 8-10, 1040 Vienna, Austria; 6 Human Evolution and Archaeological Sciences and IUAH, University of Vienna, Vienna, Austria

**Keywords:** Economic behavior, Raw material procurement, Neolithic, Western Anatolia, Çukuriçi Höyük, Geochemistry, Paleoeconomy, Statistics

## Abstract

The expansion of the Neolithic way of life triggered the most profound changes in peoples’ socioeconomic behaviors, including how critical resources for everyday life were managed. Recent research spearheaded by ancient DNA analysis has greatly contributed to our understanding of the main direction of Neolithisation spreading from western Anatolia into central Europe. Due to the diverse processes involved in Neolithisation, which resulted in a high diversity of regional and local phenomena, the underlying mechanisms of these developments are still largely unexplored. One of these mechanisms is economic behavior and resource management. Neolithic economic behavior is the result of social processes involving the physical actions of the procurement, processing, use, discard, and distribution of raw materials as well as finished products for utilitarian needs and to create and maintain social relations. Within this continuum, the key for tracing meaningful behavioral patterns is the identification of raw material procurement. Since stone tools are among the most ubiquitous and stable finds at Neolithic sites, they are ideally suited for this endeavor. Here, we present the results of a case study from the Neolithic site of Çukuriçi Höyük in western Anatolia tracing lithic raw material procurement. We employ a novel approach using geochemical provenance analyses coupled with quantitative technological and econometric methods. The key finding of this diachronic study covering almost 700 years revealed patterns of socioeconomic dynamics undetectable through conventional analytical approaches. We demonstrate that technological concepts fluctuate over time and are subject to innovations, whereas raw material procurement remains a stable element.

**Supplementary Information:**

The online version contains supplementary material available at 10.1007/s10816-024-09681-6.

## Introduction

Neolithisation, *i.e.*, the dispersion of the Neolithic way of life, is one of the most significant transformative processes humanity has undergone. From a European perspective, this development commenced approximately 6700 BC in west Anatolia and was all but straightforward, covering a timespan of roughly 1000 years (*e.g.*, Horejs *et al.,* 2015; Krauß, 2011; Özdoğan *et al.,* 2012). The cultural, social, and economic changes related to the adoption of sedentism, farming and herding in contrast to hunting and gathering ultimately shaped a distinct Neolithic mindset. With the transition to a sedentary lifeway, targeted resource management and storage gained increasing importance since, in contrast to mobile hunter-gatherers, sedentary communities were forced to ensure a more efficient, stable, and continuous supply of resources. These requirements had implications on how raw materials were procured, with a greater emphasis on planned acquisition strategies and stockpiling rather than the opportunistic, embedded procurement typical of more mobile groups. Sedentary Neolithic communities engaged in farming, herding, and fishing developed new technologies associated with these subsistence strategies, which also reduced the reliance on opportunistic raw material procurement. Instead, raw material acquisition became more organized and specialized, including exchange and distribution networks, which were established to build and maintain social relations and acquire specific goods. In short, the Neolithic mindset shifted towards a more targeted economic behavior than in previous periods (*e.g.*, Bogaard, 2005; Kuijt, 2000a, b; Rosenberg, 1998; Sherratt, 1981).

The resulting deeply rooted behavioral patterns are reflected in various ways in the archaeological record, particularly site architecture and material culture. Although ancient DNA and isotope analyses were able to demonstrate that the main Neolithic trajectories were oriented from the southeast to the northwest (*e.g.*, Hofmanová *et al.,* 2016; Lazaridis *et al.,* 2022; Marchi *et al.,* 2022), the complex mechanisms underlying this expansion are still not fully understood. The most crucial questions in this regard concern socioeconomic strategies, adaptations, and landscape use of the early farmers and herders.

Reconstructing these aspects of Neolithic socioeconomic behavior at the point of departure, in this case western Anatolia, is the foundation for understanding the diversity of economic systems that emerged among early agro-pastoral communities in the Balkans and Central Europe. Within the archaeological record, economic behavior can be traced by analyzing resource management strategies from a diachronic perspective to reveal dynamic processes at play within economic frameworks. One crucial aspect is using materials that allow for such an endeavor. Regarding resource management, chipped stone tools are among the best suited due to their wide use, abundance, and durability at prehistoric and specifically Neolithic sites.

**Fig. 1:**
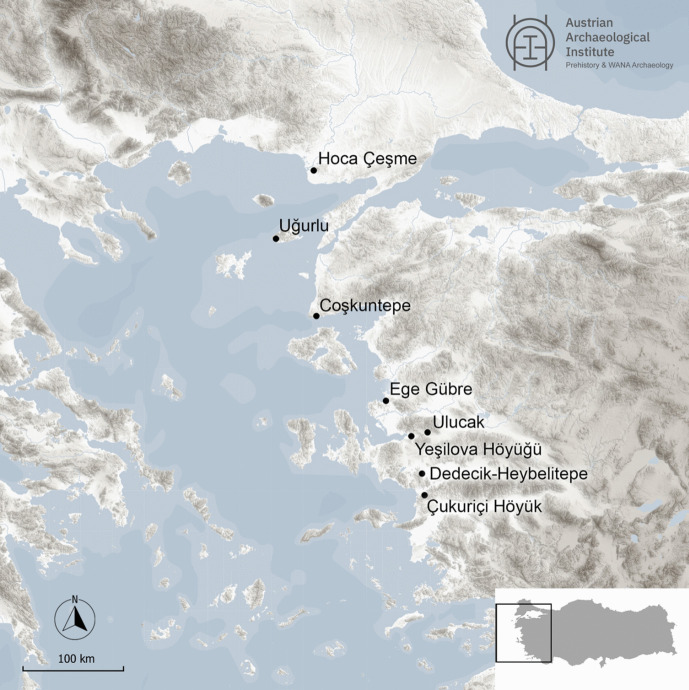
Location of Çukuriçi Höyük and other Neolithic sites in westernmost and Aegean Anatolia. Graphic: M. Börner ÖAI/ÖAW

Here, we present the results of an integrated analytical case study investigating diachronic lithic resource management, more precisely chert economy, at the Neolithic site of Çukuriçi Höyük in western Anatolia. Çukuriçi Höyük is a coastal tell site situated in the hinterland of the ancient city of Ephesus. Today, the tell is deeply covered by alluvium and only visible as a shallow mound; however, recent excavations revealed an overall height of the cultural layers of 7 m covering an area of at least 200 × 160 m equating to 32,000 m^2^ (Stock *et al.,* 2013). Following initial investigations in 1995, systematic excavations were conducted between 2006 and 2014 (with a break in 2010) by B. Horejs and her team,^1^ which uncovered settlement traces from the Early Neolithic up to the Early Bronze Age (Horejs, 2012, 2016, 2017; Horejs *et al.,* 2011). The Neolithic phases cover a settlement history of ca. 700 years, which places the site among a series of comparable western Anatolian Neolithic tells, *i.e.*, Ulucak, Ege Gübre, Yesilova, and Dedecik Heybelitepe, of which Çukuriçi and Ulucak represent the earliest Neolithic pioneer sites (*e.g.*, Çilingiroğlu *et al.,* 2012; Derin, 2012; Lichter & Meriç, 2012; Sağlamtimur, 2012; Fig. 1). These sites represent the Neolithic occupation phase along the central Aegean coast known today (Galik & Horejs, 2011, 89), which is why all of them can be regarded as key sites for studying the Neolithisation of western Anatolia and subsequent developments.

Altogether, six sequential Neolithic settlement phases (ÇuHö XIII-VIII from oldest to youngest) were recognized at Çukuriçi Höyük and dated according to contextualized material culture remains and a series of over 100 radiocarbon dates (Table 1).

**Table 1:** Çukuriçi Höyük settlement phases with periodization and preliminary absolute dating. Adapted from Horejs, 2017, Fig. 1.5. Graphic: M. Brandl ÖAI/ÖAW

Settlement Phase	Archaeological Period	Dating
ÇuHö VIII	Late Neolithic	c. 6200 - 5970 calBC
ÇuHö IX	Late Neolithic	c. 6300 - 6200
ÇuHö X	Late Neolithic	c. 6400 - 6300
ÇuHö XI	Late Neolithic	c. 6500 - 6400
ÇuHö XII	Early Neolithic	c. 6600 - 6500 calBC
ÇuHö XIII	Early Neolithic	c. 6680 - 6600 calBC

### Lithic Resource Management and Chert Economy

This study is situated in a Neolithic context, during which we think the concept of capitalist market economy in its modern form did not yet exist, and to date, there is no evidence of physical marketplaces before the Bronze Age in the Aegean (Alberti, 2016; Ialongo *et al.,* 2021; Parkinson *et al.,* 2013, 413). This is not to say that there was no exchange between groups and individuals to acquire specific objects and fulfil utilitarian needs; however, although profit making certainly also had its place in this economic setting, it might not always have been the guiding principle. After a long period of discord (including the so-called formalist-substantivist debate; *e.g.*, Baron & Millhauser, 2021, 2; Wilk & Cliggett, 2018, 12), economic anthropologists largely agree that exchange and distribution of exogenous objects within early farming economies were initiated and maintained to satisfy two intertwined objectives: to establish and sustain social relations through networking and to obtain resources for utilitarian needs (*e.g.*, Binder, 2008; Nazaroff *et al.,* 2016; Pétrequin *et al.,* 2013, 78; Perlès, 2001, 3). These aspects cannot be separated without misconstruing premodern economic concepts, which are therefore better referred to as socioeconomic. Consequently, the key organizing principles for resource distribution and exchange in such socioeconomic systems are reciprocal arrangements between individuals and communities (Binder, 2008; Carter, 2016; Graeber, 2011, 29–32; Nowak, 2015; Pétrequin *et al.,* 2013; Wilk & Cliggett, 2018, 163).

We propose one analytical pathway to the investigation of economic behavior within this framework by tracing the structure of sequenced actions underlying resource management strategies, which entail the procurement, use, and distribution of particular materials. Within this continuum, the core issue at hand concerns the determination of potential procurement modes of lithic resources. During recent decades, various approaches to tackle this issue have been proposed (*e.g.*, Allard & Denis, 2021; Andrefsky, 1994; Brantingham, 2003; Ericson, 1984; Garvey, 2015; De Grooth, 2007; Perlès, 1992, 2001; Pop, 2016; Rosenberg *et al.,* 2021; Soto *et al.,* 2018). However, there exist only a few studies explicitly dealing with Neolithic non-obsidian procurement strategies from the wider circum-Mediterranean area.

The majority of earlier studies sourcing lithic raw materials in Anatolia were clearly oriented towards obsidian (*e.g.*, Bigazzi *et al.,* 1998; Carter *et al.,* 2006, 2008, 2011, 2013; Carter & Shackley 2007; During & Gratuze 2013; Gratuze, 1999; Milić, 2014; Perlès *et al.,* 2011; Poupeau *et al.,* 2010; Keller & Seifried 1990). This can be explained by the challenges associated with chert analysis, which are more complex than those for obsidian. The main issue in this regard was the lack of adequate methods for characterizing and determining the origin of chert raw materials (*e.g.*, Nazaroff *et al.,* 2013, 343). As a result, chert and other knappable raw materials remain significantly underexplored in the region (*e.g.*, Bergner *et al.,* 2009, 251). Nevertheless, attempts to determine the provenance of cherts in archaeological contexts were undertaken, *e.g.*, by Pawlikowski (2002) for the region of Antalya, and several researchers in the framework of projects related to Çatalhöyük (*e.g.*, Bezic, 2007; Doherty *et al.,* 2007). The most extensive work in this regard was presented by Nazaroff *et al.* (2013; 2015) in the framework of the Anatolian Archaeological Raw Material Survey (AARMS) project applying X-ray fluorescence (XRF). More recently, Ostaptchouk (2020) used FTIR to obtain potential provenance information for chert and jasper raw materials in the surroundings of Çatalhöyük.

For the current study, we employed laser ablation inductively coupled plasma mass spectrometry (LA-ICP-MS) to detect trace element concentrations that can be used to reveal the provenance of archaeological materials when compared to geological samples from identified outcrops. Various interdisciplinary techniques have been applied to chert and flint sourcing in the past (*e.g.*, Wright, 1967; Aspinall & Feather, 1972; Reed, 1990; Poolton *et al.,* 1995; Gratuze, 1999; Gratuze *et al.,* 2001; Pillay, 2001; Thacker & Ellwood, 2002; Tykot, 2003, 2004; Cinta Pinzaru *et al.,* 2008; Parish, 2009), of which geochemical approaches such as neutron activation analysis (NAA; *e.g.*, Glascock & Speakman 2008), X-ray fluorescence (XRF; *e.g.*, Rafferty *et al.* 2007; Hughes *et al.,* 2010), and LA-ICP-MS (*e.g.*, Roll *et al.,* 2005; Speakman & Neff, 2005; Speakman *et al.,* 2007) produced the most promising results.

Given the heterogeneous nature of micro- and cryptocrystalline siliceous rocks and their typically very low trace element concentrations (Luedtke, 1992; Gratuze *et al.,* 2001; Speakman *et al.,* 2002), LA-ICP-MS is increasingly utilized for their chemical characterization (Delage, 1997; Evans *et al.,* 2007; Roll *et al.,* 2005; Morgenstein, 2006; Speakman *et al.,* 2007; Moroni & Petrelli, 2005; Moreau *et al.,* 2016). LA-ICP-MS requires minimal sample preparation and enables the rapid, simultaneous detection of up to 50 elements with high precision (Neff, 2012; Resano *et al.,* 2010; Sánchez de la Torre *et al.,* 2017; Speer, 2014).

## Underlying Assumptions

When discussing lithic resource management and specifically procurement, one crucial question concerns unlimited versus limited source access. The assessment of potential resource control requires a close examination of the larger socioeconomic framework of a particular society. For the Anatolian and the Aegean Neolithic, it has been demonstrated that strict resource control involving access restrictions to sources did not play a significant role in the litho-economic strategies of the early farmers and herders (Torrence, 1984, 1986). One of the most convincing indicators that even particularly coveted materials were not monopolized during the Aegean Neolithic is the case of obsidian from Melos, which appears to have been freely available until the Early Bronze Age (Cherry & Torrence, 1982; Kakavakis, 2015, 36; Torrence, 1984; Torrence, 1986, 198–216). Therefore, we can postulate that lithic raw material sources of western Anatolian Neolithic sites such as Çukuriçi Höyük were in all likelihood exploited without any severe restrictions to access.

With this in mind, the following scenarios for lithic resource procurement within the specific setting of this case study are conceivable:

- Embedded procurement as part of other, predominantly subsistence, activities (*e.g.*, Binford, 1979, 1980, 1985; Gould & Saggers, 1985). Typically associated with hunter-gatherer economies, it cannot entirely be ruled out for sedentary farmers and herders (and particularly herders, since they regularly moved across the landscape with their animals).
- Direct-targeted procurement implies that people purposely went to a source to acquire materials.
- Indirect procurement requiring mediators to obtain raw materials or half-finished and finished objects, therefore also referred to as social procurement.

These procurement strategies were often flexible and adaptive (Tomasso & Porraz, 2016, 164) and could alternate for particular materials, especially from a diachronic perspective. The nuanced assessment of which mechanisms facilitated the acquisition of lithic objects made from various raw material types is therefore crucial for the interpretation of otherwise imperceptible behavioral patterns.

As stated earlier, various approaches attempted to address this issue; however, all hypotheses and models proposed for prehistoric realities are necessarily based on assumptions which cannot be objectively verified. As Mears and Wilson (2023, 14) conclude, “*we have no way of testing for intention: we know what they (prehistoric people) did, not what they meant to do*.”

We agree that we will never be able to reveal intention; however, we are optimistic that we can elucidate aspects of human behavior, in this case economic. To this end, our contribution builds upon and expands previous endeavors that explore the nature and underpinnings of Neolithic stone tool economy, *i.e.*, that of fully sedentary farming societies through several systematic lines of investigation. Using key elements and fundamental assumptions from this previous research, we build a theoretical framework to assess potential procurement strategies. Subsequently, we model the results from lithic raw material analyses and technological examinations within this theoretical framework to reveal the most likely by excluding the most unlikely possibilities. In that, semi-quantitative assessments can be made and behavioral patterns behind economic actions can be interpreted in “best fit” scenarios for procurement strategies in accordance with the available archaeological data. In tandem with the high quality and resolution of our analytical results, the model we propose goes beyond the scope typically examined to answer questions related to lithic raw material procurement.

The assumptions the theoretical framework of this case study is based upon, including the reasoning behind them, are detailed as follows:

### Assumption #1. There Existed a Local Supply Zone Around Each Neolithic Settlement or Village

Inspired by the concept of site catchment analysis (Roper, 1979; Vita-Finzi & Higgs 1970), the local supply zone or immediate exploitation territory is defined as “that area which was accessible to habitual exploitation by the occupants of the site” (Jarman *et al.,* 1982, 32; also: Allard & Denis, 2021, 8–10; Bakels, 1982; Dubouloz *et al.*, 2012). For sedentary farming and herding societies, a maximum of 10 km around a settlement corresponding to 2 or 3 h of walking time (depending on the physiography of the landscape) is assumed for this local supply zone based on ethnographical accounts (Jarman *et al.,* 1982, 32).

Although we do not assume strict resource control for the Aegean Neolithic as discussed above, it is expected that materials from within the immediate exploitation range of a community were not exploited by members of other groups, but passed on in the course of “repeated reciprocal exchanges” with the principal aim to establish and maintain social relations through networks (*e.g.*, Perlès, 1992, 116–117). All materials from within this supply zone are classified as “local.”

### Assumption #2. For Materials Outside of the Local Supply Zone, Indirect Procurement Is the More Likely Scenario, and Direct Procurement Is the Exception in Sedentary Communities

Consequently, it is conceivable that sedentary Neolithic communities acquired materials from outside of the local supply zone more likely through social networks than in the course of direct procurement (Allard & Denis, 2021; Féblot-Augustins & Perlès, 1992; Perlès, 1992, 116–117). This assumption does not imply the simple equation that local materials were always directly procured, and non-local materials indirectly. Although some ethnographic accounts support this notion (*e.g.*, Féblot-Augustins & Perlès, 1992; Torrence, 1986), this is only one element to be considered and not an exclusive parameter that allows for the evaluation of a procurement mode. It needs to be considered in tandem with the technological skill reflected in morphological standardization of the primary target products and raw material quality.

### Assumption #3. Objects That Were Passed on Through Social Networks Display a Higher Degree of Morphological Standardization Than Directly Acquired Items

A certain degree of “standardization” or maybe better “uniformization” can be observed, when we assume that objects exchanged through social networks (*e.g.*, cores or blade blanks) tended to be produced by craftspeople at the higher end of the skill spectrum (*e.g.*, Torrence, 1986, 43–46). According to Pelegrin (1990, 117), elaborate knapping activities result in “standardized knapped or trimmed products,” which can be recognized through the low metric variation in the size of the stone tools and the symmetry of their cross section (Allard & Denis, 2022, 24; Bamforth & Finlay, 2008). Directly procured materials—except such of particular value—were potentially worked by knappers who possessed varying levels of skill representing a cross section of the population from a village, which is reflected in a larger morphological variety amongst the target products (*e.g.*, Allard & Denis, 2021, 15; Perlès *et al.,* 2011, 47; Torrence, 1986, 44–45).

### Assumption #4. Objects That Were Indirectly Procured Show a Higher Degree of Transformation

Only very selected elements of the chaîne opératoire are passed on through social networks, whereas a more diverse spectrum is expected when raw materials are directly collected or quarried (*e.g.*, Allard & Denis, 2021, 13–15; Kakavakis, 2015, 38–40). If this applies, the degree of transformation of the objects brought into the site is generally higher for imported materials (*e.g.*, Perlès *et al.,* 2011, 47).

### Assumption #5. Indirectly Acquired Materials Tend to Be of High-Quality and Occur in Lower Numbers Than Objects Made from Directly Procured High-Quality Material

Although the goal of raw material procurement is always to obtain the material most suitable for a specific task, the choice is limited by the geological conditions within the local supply zone of a settlement. The solution is at hand: Either one has to invest energy and undertake targeted expeditions to sources producing materials of suitable quality, or one obtains such materials through exchange. High-quality materials that occur only in low numbers compared to other high-quality materials in the assemblage and fulfil the criteria of assumptions #3 and #4 can be suspected of having been acquired in a different way than those displaying a different way than such displaying a higher morphological and transformative variation and occurring in higher amounts (*e.g.*, De Grooth, 2007; Kakavakis, 2015, 38–40; Perlès, 2001, 207–208).

### Assumption #6. When Cores Were Systematically Reduced and Pre-formed Off-Site, Direct or Indirect Procurement Are More Likely Scenarios Than Embedded Procurement

It can be argued that raw materials consistently appearing in a pre-worked state suitable for subsequent specialized production reflect the deliberate action of knappers who knew exactly what they were doing (*e.g.*, Pelegrin, 1990; Perlès *et al.,* 2011, 47). Although the absence of initial core reduction debris of particular raw materials in an assemblage does not preclude embedded procurement, scenarios in which cores were frequently pre-shaped off-site are more likely the result of activities by skilled knappers engaged in deliberate procurement, either direct or indirect. From this perspective, strongly pre-worked and at the same time morphologically standardized lithic tools, predominantly made from high-quality raw materials that originate from outside of the local supply zone of a settlement and occur in low numbers when compared to other materials of similarly high quality in the lithic assemblage are assumed to have been indirectly acquired. This is not to say that embedded raw material procurement did not exist during Neolithic times; however, based on our knowledge about the general character of Neolithic socioeconomic organization, this way of raw material acquisition did very likely not constitute a significant economic factor. Simply stated, although still possible, embedded procurement is essentially not a concept of sedentary Neolithic communities (*e.g.*, Kuijt, 2000a; Rosenberg, 1998).

## Materials and Methods

From all Neolithic contexts at Çukuriçi Höyük, approximately 18,000 lithic artifacts were recovered. As indicated in Table 2, obsidian from the Aegean island of Melos^2^ (Milić, 2019a, 488–490; Schwall *et al.,* 2020, 15) becomes the dominant raw material within the chipped stone assemblage in phase XII, which immediately succeeds the pioneering phase XIII and reaches the peak of its use at the end of the Neolithic sequence in phase VIII. From phase XI on, it consistently exceeds the 80% threshold, highlighting its importance for the Neolithic community as utilitarian as well as social (and perhaps prestigious) objects (Milić, 2019a, 489–490).

**Table 2:** Composition of the chipped stone assemblage from Çukuriçi Höyük according to settlement phases. The chert artifacts used for this study are listed separately. Adapted from Milić, 2019a, 489, Fig. 2. Graphic: M. Brandl—B. Milić ÖAI/ÖAW

Çukuriçi Höyük chipped stone assemblage	Early Neolithic (6680–6500 cal. BC)		Late Neolithic (6500–5970 cal. BC)			
	Phase XIII	Phase XII	Phase XI	Phase X	Phase IX	Phase VIII
Obsidian	33%	68%	80%	83%	85%	86%
Chert and other siliceous rocks	67%	32%	20%	17%	15%	14%
No. of artefacts	216	301	2859	6127	4997	3360
Total number	17.860					
Cherts artifacts in this study	109	93	40	460	96	253
Total number cherts studied	1031					

Here, we discuss the second largest group of raw materials, the chert assemblage. While it is assumed that Melian obsidian was procured directly by the seafaring community based at Çukuriçi Höyük (Milić & Horejs, 2017, 44; Schwall *et al.,* 2020, 15), which might have functioned as a gateway community for this particular raw material in coastal western Anatolia, the situation differs significantly for the various chert types identified within Çukuriçi Höyük’s lithic assemblage. As shown through technological analyses, chert and obsidian tools were produced and used differently. Obsidian technology predominantly aimed at regular blade production, while the chert assemblage displays a more diverse spectrum.

The reconstruction of Çukuriçi Höyük’s chert economy offers unique insights into microregional and regional resource management strategies indicative of the economic behavior of the community in contrast to the supraregional (*i.e.*, obsidian) perspective. The chert assemblage used for this study comprises 1031 lithics, which were characterized based on petrographic criteria and analyzed for their geologic provenance and specific technological criteria suitable for lithoeconomic calculations. The sample was selected based on relevant contexts (house complexes and use horizons) within each settlement phase (Table 2).

## Analytical Protocol

**Fig. 2:**
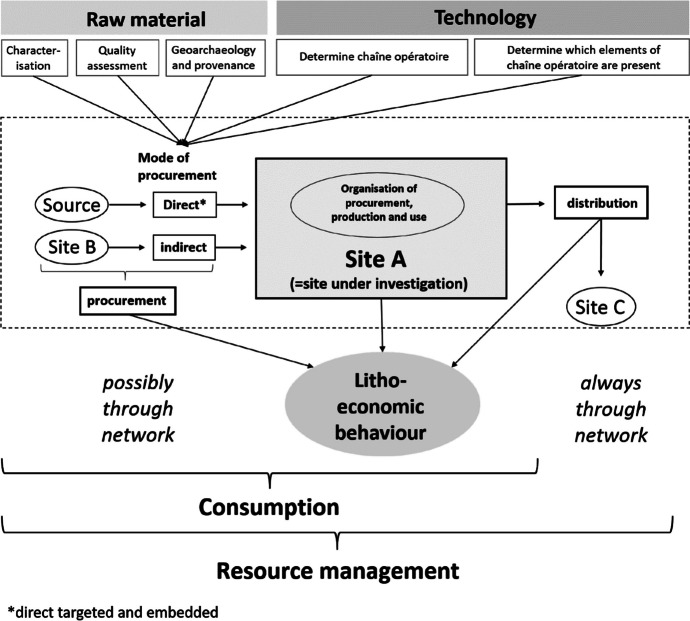
Analytical protocol used for this study to reconstruct lithic raw material procurement within the framework of Neolithic resource management. Graphic: M. Brandl ÖAI/ÖAW

Within the concept of lithic economy, we propose here that resource procurement and use (*i.e.*, the production, practical application, and discarding of lithic objects) constitute the element of consumption. Consumption entails the choices people make regarding raw material selection and technological aspects (*e.g.*, Hughes, 2011; Nazaroff *et al.,* 2013, 2014; Torrence, 1986). If a settlement acts as a mediator within a network, resource distribution completes the spectrum of resource management. Tracing the procurement element within this socioeconomic framework requires qualitative and quantitative assessments of the investigated materials, including their full characterization, quality assessment, provenance analyses, and technological studies aiming at the reconstruction of the individual chaîne opératoire for each individual raw material type (Fig. 2). As such, we take a site-centric approach to study lithic resource management; *i.e.*, the central analytical unit is the lithic assemblage from a particular site (Tomasso & Porraz, 2016, 166), in this case Çukuriçi Höyük.

For the raw material aspects of this study as outlined in Fig. 2, we apply the multi layered chert sourcing approach (MLA) specifically developed for in-depth characterization and provenance analyses of siliceous rocks (Brandl, 2016; Brandl *et al.,* 2018). Technological examinations of all lithic artifacts were carried out by B. Milić, reconstructing the chaîne opératoire of the chipped stone industry at Çukuriçi Höyük (Milić, 2018, 2019a), which is the foundation for our approach applying lithic transformation analysis as outlined below.

### Raw Material Characterization and Grouping

Lithic raw material characterization follows terminological and petrographic criteria defined by Brandl (2014) to achieve visually coherent groups based on color, rock texture, cleft frequency, and granularity. The resulting preliminary raw material groups were subsequently tested for their consistency through microfacies analysis.

Stereomicroscopic microfacies analysis identifies the microstructure of siliceous rocks focusing on the size, shape, and spatial arrangement of rock-building components, as well as inclusions and bioclasts. The latter are particularly useful for determining the age and depositional environment (the facies) of organogenic SiO_2_ modifications such as chert or flint. For nondestructive microscopic examination, we used a Leica M125 reflected light stereomicroscope equipped with a DFC295 camera system to document unpolished rock surfaces under water immersion for enhanced optical performance, a standard petrographic procedure for microfacies applications in archaeometry (Affolter, 2002; Brooks, 1989, 53–71; Přichystal, 1984, 146–152). Through the combination of macroscopic and microscopic criteria, consistent type varieties (chert var. 1_1 to 1_999) were established within the chert assemblage, for each of which the individual quality was assessed.

### Quality Assessment

Raw material quality is an essential aspect for objectively comparing different lithic materials within a lithic assemblage, influencing various aspects of the production mode (*e.g.*, Andrefsky, 2009, 77). Quality, however, is not a universally measurable constant. For lithic industries, it has to be evaluated from the perspective of the desired target products. Different technological systems require different materials, which is why “quality” can significantly vary considering the diverse technological requirements, *e.g.*, core tools versus thin bladelets and blades. As such, without applying more sophisticated analytical techniques such as fracture toughness (Woods, 2011) or hardness (Marshall *et al.,* 1982), comparable parameters for assessing the knapping quality of siliceous rocks include the rock texture (*i.e.*, bioclasts and inclusions, degree of silicification), and the frequency of naturally occurring clefts from tectonic stress of the host rock. Consequently, the resulting quality assignment of raw material varieties as high, medium, or low in an assemblage is a relative value and is only comparable with assemblages from other sites if the same parameters are considered.

### Geoarchaeology and Provenance

One of the fundamental aspects for tracing lithic resource management from the site perspective concerns the geological origin of the studied lithic materials. On the most fundamental level, provenance information is used to differentiate between local and nonlocal materials in the assemblage. This differentiation is crucial for lithoeconomic investigations. We conducted geoarchaeological surveys based on the detailed geological map from Çakmakoğlu (2007) systematically documenting available raw materials for chipped stone production to determine the potential local supply zone of Neolithic Çukuriçi Höyük and additional promising source areas outside of this territory.

For a preliminary investigation comparing the sources of various rock types found at the site, we used a model designed for a generalized comparison (Schwall *et al.,* 2020, 2).

In the current study, we were able to refine this model and evaluate the local supply zone for chert independent of predefined distance ranges based on the results of our geoarchaeological surveys. Systematic geoarchaeological surveys covered an area of approximately 10–15 km around the site and were subsequently extended further towards the north into an area rich in lacustrine siliceous rocks and previously suggested to be a potential source region of many chert varieties found in western Anatolian Neolithic sites, namely, Ege Gübre (*e.g.*, Sağlamtimur, 2012).

As explained in more details below, the survey showed that three lacustrine chert deposits exist within the intensively investigated 15-km range, of which only one could be identified as the source of raw materials used for chipped stone production. The other two deposits within this area were not accessible during Neolithic times as their position and absolute lack of any chipping debris in their surrounding suggests. Since in the south and east of Çukuriçi Höyük no chert-bearing geological formations occur, we focused on the closest known source area of lacustrine chert outside of the immediate surroundings of the site, which can be found 90 km to the north. Within the 15-km range, geological samples were collected as a reference for comparison with the archaeological material in our provenance study. However, samples from outside of our immediate study area could only be investigated on-site. Consequently, we cannot provide geochemical data from these sources but have to rely on petrographic comparisons with the chert from the studied assemblage.

### Geochemistry

In some cases, microfacies determination can inform us about the potential provenance of lithic rocks by narrowing down a distinct source area. This, however, is only possible for very advantageous geological settings. In the case of Çukuriçi Höyük, geochemistry had to be employed to test hypotheses relating to local versus nonlocal chert types. For this task, selected artifacts from the established microscopic type varieties were analyzed with laser ablation inductively coupled plasma-mass spectrometry (LA-ICP-MS) at the Department of Earth Sciences—NAWI Graz Geocenter, University of Graz, Austria. An ESI NWR193 laser unit coupled with an Agilent 7900 ICP-quadruple mass spectrometer was used for these analyses. Ablation was performed with a 193-nm laser pulsed at 7 Hz, a spot size of 75 μm, and an energy of ~ 5 J/cm^2^. Helium, the carrier gas, was established at ~ 0.8 l/min flow, and data were acquired in time-resolved mode. The standard glass NIST612 was routinely analyzed for standardization and drift correction, while the reference glass BCR-2G was analyzed as an unknown and reproduced better than 10% relative error. Silicon (Si) was used as the internal standard.

Altogether, 15 geological samples representing the variability of the chert found at Çanakgöl Tepe, the only source within the immediate supply zone of the site, and 44 archaeological artifacts were placed into epoxy resin mounts and polished prior to analysis. To control naturally occurring heterogeneities in sedimentary siliceous rocks such as chert, a minimum of three (in some cases four) particular spots were analyzed on each sample.

### Statistical Data Treatment

The geochemical results were statistically evaluated using bivariate scatter plots for initial assessments and source-specific information. Subsequently, we applied robust principal component analysis (rPCA) coupled with linear discriminant analysis (LDA) to achieve optimal group assignment indicative of the provenance of particular chert types and to substantiate the geochemical findings.

### Transformation Analysis (TS)

Once the provenance of the lithic artifacts is established and local versus nonlocal resources are defined based on geoarchaeological investigations and material analyses, we include information about the individual chaîne opératoire for each chert type. Potential information regarding the mode of procurement can be gathered by reconstructing in which state of transformation a work piece was introduced into an archaeological site, *i.e.*, how heavily worked the raw piece was upon introduction. Within the chaîne opératoire concept, this can be best accomplished by lithic transformation analysis following Roebroeks (1988) and further developed by, *e.g.*, Uthmeier (2004) and Nigst (2012).

For our current study, we define five distinct transformation stages (TS) of workpieces:

- TS1: Raw and unworked pieces.
- TS2: Tested and pre-worked pieces and early-stage reduction debris (ESR).
- TS3: Rejected cores and late-stage reduction debris (LSR).
- TS4: Unretouched and unused target products (blanks).
- TS5: Exhausted cores and discarded tools (retouched and unretouched).

This sequence covers all possible working stages of lithic reduction. The relation between lithic production debris from reduction on-site and the degree of processing of workpieces before they were brought into the settlement, visible through the lack of specific TS, is expected to potentially reveal patterns indicative of particular modes of resource procurement. This requires identifying the traceable key elements distinguishing the three potential procurement modes defined above (Table 3). Meaningful patterns indicative of procurement behavior however can only be traced when directly comparing the results of TS analysis for all raw material varieties in the assemblage. When some clearly stand out from the others, *e.g.*, by exclusively displaying the latest TS stages, and in combination with the results of the other strands of analysis, this can indicate that these materials might have been differently acquired.

**Table 3:** Criteria for the assessment of potential different modes of raw material procurement. Graphic: M. Brandl ÖAI/ÖAW

Line of investigation	Paramaters	Potential procurement mode		
		Embedded	Direct	Indirect
Raw materials in lithic assemblage	main provenance	local	local	non-local
	location of sources	wider supply zone	immediate supply zone	outside of supply zone
	abundance	variable	high	low
	raw material quality (RMQ)	variable	variable	high
	selectivity of sources and materials	low	high	very high
Technological aspects	degree of standardization (i.e. morphological variation) of blanks when procured	low	low	high
	transformation stages (TS) present	1 – 5	1 – 5	3 – 5

Embedded procurement is characterized by the dominance of local materials displaying a low degree of selectivity in both raw material types and their quality, and a significant degree of randomness can be observed (Adams & MacDonald, 2015; Binford, 1979). This procurement mode is only determinable through the examination of the overall variability of raw materials within a lithic assemblage.

Direct procurement also predominantly relies on local materials; however, since this acquisition mode represents a targeted action, the degree of randomness decreases in favor of deliberate choices and raw material selection. Distance-decay patterns become an observable factor. For quality, this factor must be viewed from the perspective of target production (*e.g.*, Close, 1999; Newman, 1994). On-site reduction debris can reach high volumes, while the standardization of preforms brought into the site is generally expected to be low.

Indirect procurement is usually associated with nonlocal materials unless restrictions of source access exist within the local supply zone of a site, which we do not assume for the Anatolian and Aegean Neolithic as outlined in the beginning. In a lithic assemblage, indirectly procured materials are characterized by a combination of generally low numbers of artifacts made from materials derived from sources outside of the “local” supply range of a community, which were additionally heavily pre-worked upon introduction into the archaeological site and display a high degree of standardization. The quality of the materials is typically situated at the higher end of the continuum (*e.g.*, Allard & Denis, 2021, 13–17; Parow-Souchon & Purschwitz, 2020; Tomasso & Porraz, 2016, 164). While these criteria could also indicate direct procurement of curated materials by more mobile groups, this is rather unlikely for Neolithic sedentary farming and herding communities.

## Results

### Characterization and RMQ

Within the analyzed chert assemblage, which comprises 1031 lithics, 11 distinct varieties were identified based on macroscopic (visual) pregrouping with subsequent stereomicroscopic microfacies information (Table 4). This assessment also includes an evaluation of raw material quality combining granularity with cleft frequency of each chert type, parameters that can be objectively measured without the need for instrumental techniques.

**Table 4:** Results of visual grouping and microfacies analysis of the chert assemblage. Graphic: M. Brandl ÖAI/ÖAW

Raw material	Variety	No. lithics	Color acc. to Munsell Rock Color Chart 2009	Transparency*	Geol. age	Depositional facies	Texture acc. to Dunham 1962	Natural surface remains	Raw material quality	
chert	1_1	91	10R 4/2 Grayish Red – 10R 3/4 Dark Reddish Brown – 10R 2/2 Very Dusky Red	n.t., rarely s.t.	Neogene	lacustrine	wackestone	primary	high	**hq**
chert	1_1a	2	10R 2/2 Very Dusky Red – 5YR 2/2 Dusky Brown	n.t.	Neogene	lacustrine	grainstone	primary	medium	**mq**
chert	1_2	127	5YR 6/4 Light Brown – 5YR 5/2 Pale Brown – 5YR 5/6 Light Brown	s.t.	Neogene	lacustrine	mudstone - wackestone	primary	high	**hq**
chert	1_2a	36	5YR 5/6 Light Brown – 5YR 4/4 Moderate Brown	s.t.	Neogene	lacustrine	wackestone - packstone	primary	high	**hq**
chert	1_3	227	5YR 7/2 Grayish Orange Pink – 5YR 6/4 Light Brown – 5YR 5/2 Pale Brown – 5YR 5/6 Light Brown – 5YR 4/4 Moderate Brown – 5Y 3/2 Grayish Brown – 5YR 3/4 Moderate Brown	n.t.	Neogene	lacustrine	mudstone - packstone	primary	low	**lq**
chert	1_4	218	5PB 7/2 Pale Blue – 5YR 8/1 Pinkish Gray	s.t.	Neogene	lacustrine	mudstone - wackestone	primary	high	**hq**
chert	1_4a	103	N9 White – 5YR 8/1 Pinkish Gray - 5B 7/1 Light Bluish Gray	s.t.	Neogene	lacustrine	mudstone	primary	high	**hq**
chert	1_5	21	10R 3/4 Dark Reddish Brown – 10R 2/2 Very Dusky Red	n.t.	Neogene	lacustrine	packstone - grainstone	indet	medium	**mq**
chert	1_6	7	5PB 7/2 Pale Blue – 5PB 5/2 Grayish Blue	n.t.	Neogene	lacustrine	wackestone - packstone	indet	high	**hq**
chert	1_7	21	N9 White – 5YR 8/1 Pinkish Gray	n.t.	Neogene	lacustrine	mudstone - wackestone	indet	medium	**mq**
chert	1_8	25	5Y 8/1 Yellowish Gray with yellow or reddish veins	n.t.	Neogene	lacustrine	mudstone	indet	high	**hq**
chert	1_999	153	various colors	*n.a.*	Neogene	lacustrine	*n.a.*	*n.a.*	*n.a.*	

**Fig. 3:**
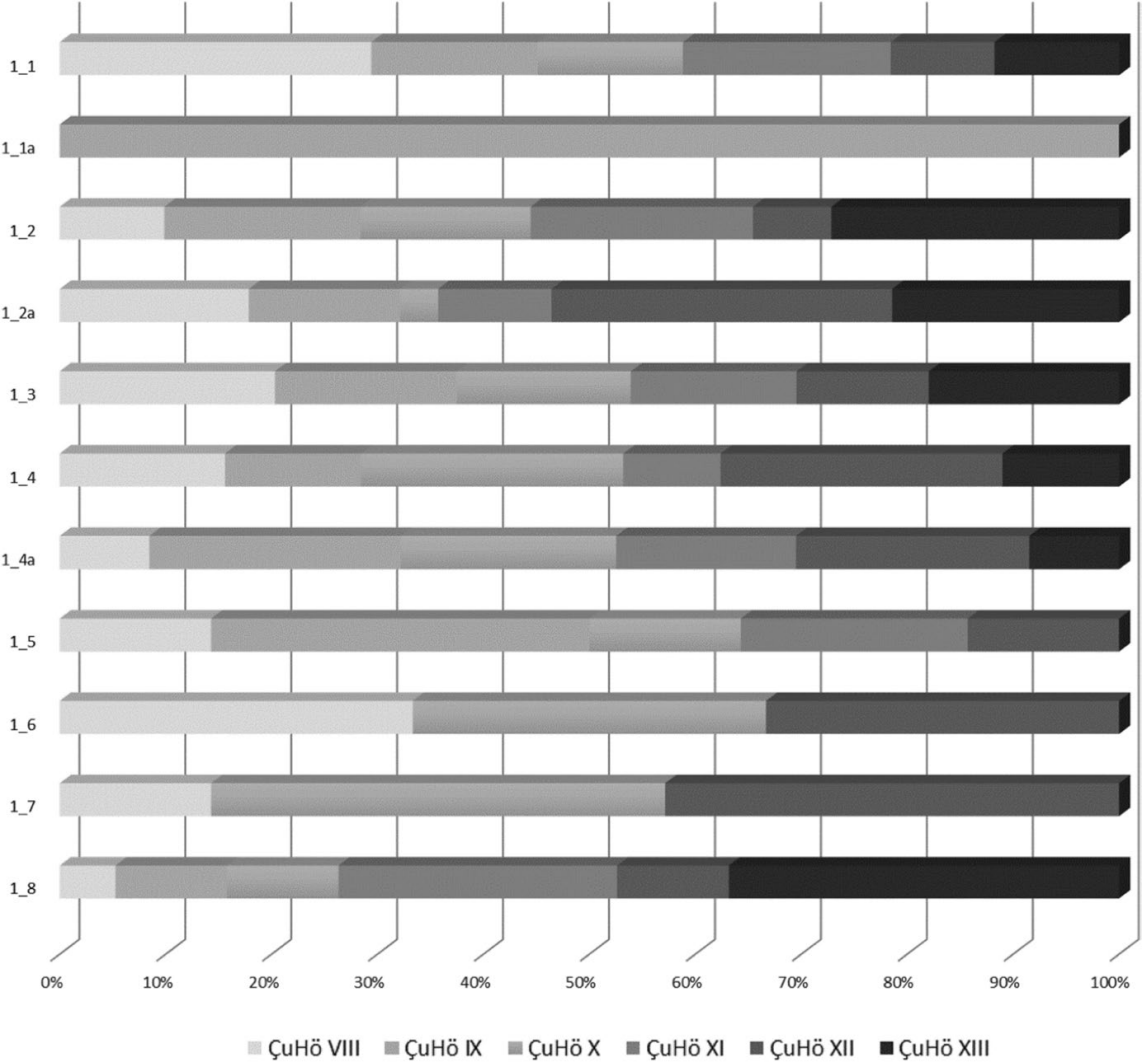
Distribution of chert varieties within Çukuriçi Höyük’s Neolithic settlement phases. Graphic: M. Brandl ÖAI/ÖAW

The proportional distribution of all chert varieties within all Neolithic phases shown in Fig. 3 illustrates that not every documented type is present in each Neolithic settlement horizon. This however supports the notion that over time we see an intensification of the use of different chert types within the assemblage.

### Provenance Analyses

**Fig. 4:**
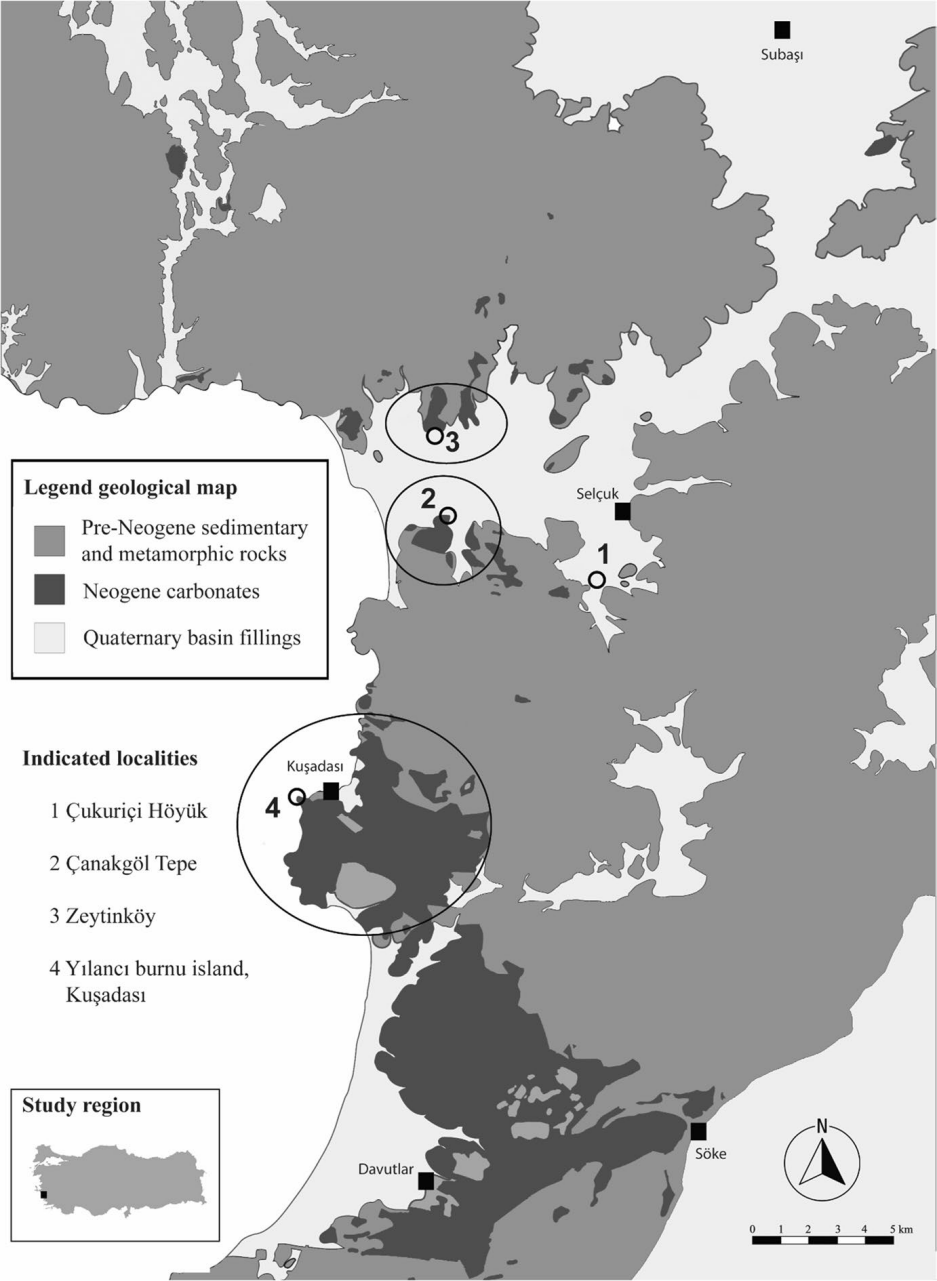
Simplified geological map of the catchment area of Çukuriçi Höyük (No. 1 in map). The chert-bearing parts of the Miocene lacustrine Kuşadası Formation with distinct outcrops (No. 2–4) are highlighted. Black squares indicate larger towns in the area. Adapted from Çakmakoğlu, 2007, Fig. 1a. Graphic: M. Brandl ÖAI/ÖAW

Our geoarchaeological examinations produced evidence for potential sources of lacustrine cherts macroscopically corresponding well to the most frequent types in Çukuriçi Höyük’s chert assemblage. These sources occur on both margins of the Küçük Menders Graben within a distance of up to 13 km from the site and belong to the Miocene Kuşadası Formation (Fig. 4). Identified source locations are Çanakgöl Tepe approximately 6 km west of the archaeological site, at Yılancı burnu island at Kuşadası ca. 13 km to the southwest, and at the village of Zeytinköy ca. 9 km to the northwest. Of these, only Çanakgöl Tepe produced material that could be used for provenance analyses, since at Zeytinköy only one chert piece of unsecured geological position and traces of silicified limestone, and at Yılancı burnu only a submerged chert layer could be detected. The latter two are mentioned as chert deposits in the vicinity of the site; however, only Çanakgöl Tepe could be considered as a likely candidate to be tested. No chert sources were detected in the region further south of Kuşadası.

Consequently, the focus of our geochemical provenance analyses was placed on Çanakgöl Tepe, the chert deposit closest to Çukuriçi Höyük. Although typically it would be desirable to analyze all geological deposits in the study area to obtain secure provenance information, there are reasonable arguments why we consider the sampling of only one source sufficient in this specific case.

Çanakgöl Tepe is the only locality that produced evidence for prehistoric chert exploitation in the form of mining debris and the presence of massive hammer stones. Additionally, due to significantly higher seawater levels during the Neolithic (Stock *et al.,* 2013, 58; Stock *et al.,* 2015, 566–567), it is almost certain that the other detected sources in this area were not accessible then because they are located close to the current seawater level, whereas the outcrops at Çanakgöl Tepe are situated on the top of a hill, which was probably an island during Neolithic times. This is supported by the fact that, in contrast to Çanakgöl Tepe, there was no evidence of chert exploitation or knapping anywhere near these other two localities.

Outside of the immediate vicinity of Çukuriçi Höyük, the closest relevant lacustrine chert-bearing limestone formations occur ca. 90 km to the north around the village of Çakmaklı and the Neolithic settlement of Ege Gübre. Chert in this region is linked to limestone of the Aliağa Formation, which forms a part of the Foça volcanic sequence.

### Microfacies Analyses

**Fig. 5:**
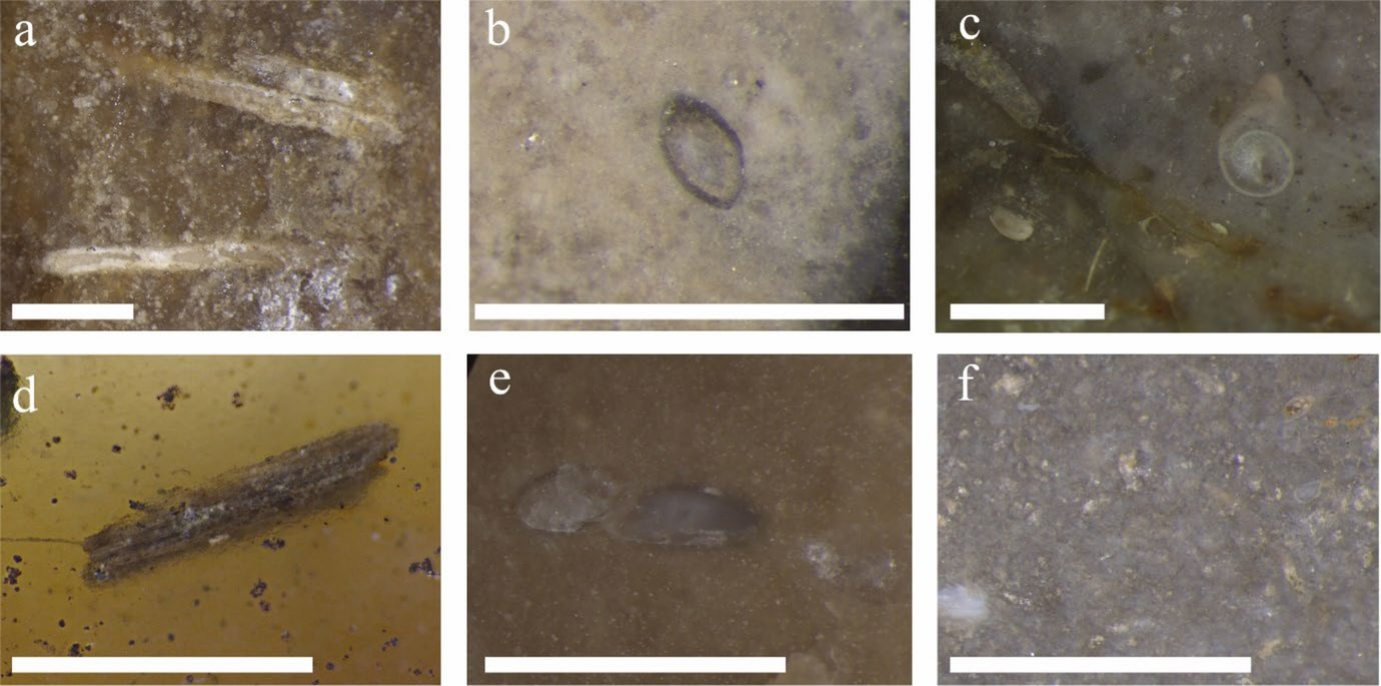
Microphotographs of chert artifacts from Çukuriçi Höyük (**a**–**d**) and geological samples from Çanakgöl Tepe (**e**, **f**). The bar corresponds to 1 mm in length. Legend: **a** and **b** chert type 1_3; **c** chert type 1_5; **d** chert type 1_2a; **e** and **f** chert type 1_3. Photos: M. Brandl ÖAI/ÖAW – M. Martinez, Amerind Foundation

Stereomicroscopic microfacies analyses confirmed a high congruence of geological samples from the Kuşadası Formation with chert types 1_1 through 1_3. All these materials are of lacustrine origin and can be found within chert layers sampled at Çanakgöl Tepe. The lacustrine nature of the cherts is confirmed through the bioclastic content, namely freshwater algae (Charophyta, Fig. 5a and d), freshwater ostracods (Fig. 5b and e), and gastropods (Fig. 5c).

The cherts from deposits within the Aliağa limestone formation display a brecciated structure, which displays similarities to the chert variety 1_8 from Çukuriçi Höyük. The latter as well as Aliağa cherts are of lacustrine origin. This area, from which it was not possible to collect samples, is the closest possible source region for this particular raw material based on our survey results and available geological information.

### Geochemistry

To test our hypothesis concerning the local lithic supply area of Çukuriçi Höyük, we analyzed 15 geological samples from Çanakgöl Tepe and 44 chert artifacts from Çukuriçi Höyük geochemically. Of the geological samples, four derive from the topmost remains of the chert formation (top-chert layer), which was almost entirely exploited during prehistoric times, and more material suitable for geochemistry could not be obtained. The other 11 samples come from a chert horizon exposed in the course of modern limestone quarrying situated at a significantly deeper position within the sequence ca. 15 m below the current surface. The archaeological sample was chosen based on petrographic analysis, with the aim of testing artifacts that showed the closest resemblance to geological material from the identified source at Çanakgöl Tepe. Consequently, chert types 1_1, 1_2, 1_2a, 1_3, 1_4, and 1_4a were selected for LA-ICP-MS analyses.

Altogether, 36 trace elements (among them REEs) were determined through LA-ICP-MS over the course of 53 (12 from the top-chert layer and 41 from the deeper horizon) individual measurements for the geological samples and 175 for the archaeological artifacts. The elements determined and used for the statistical evaluation in our study are Li7, Be9, B11, Mg24, Al27, Ca43, Ti49, V51, Cr53, Mn55, Fe56, Co59, Ni60, Cu63, Zn66, Ga71, Ge74, Sr88, Y89, Zr90, Nb93, Cs133, Ba137, La139, Ce140, Pr141, Nd146, Sm147, Eu153, Gd157, Dy163, Er166, Yb172, Pb208, Th232, and U238 (see Supplement A).

### Statistical Data Evaluation

#### Bivariate Scatter Plots

Bivariate plots using trace elements reveal source-specific conditions of the investigated materials and allow for initial assessments regarding their provenance. Generally, the presence of trace elements in the analyzed chert samples can most likely be attributed to clay minerals, which are the main carriers for *e.g.* barium (Ba), aluminum (Al), and titanium (Ti). Specific elements such as lithium (Li) and boron (B) can be employed to reconstruct the genetic environment of siliceous materials, for example, distinguishing between marine and lacustrine conditions. Taylor and McLennan (1985) reported average lithium and boron concentrations in seawater as 0.17–0.25 mg/kg and 4.5–5 mg/kg, respectively. In freshwater, the corresponding values are 0.003 mg/kg for Li and 0.01 mg/kg for B, with potentially significant variations in microenvironments.

**Fig. 6:**
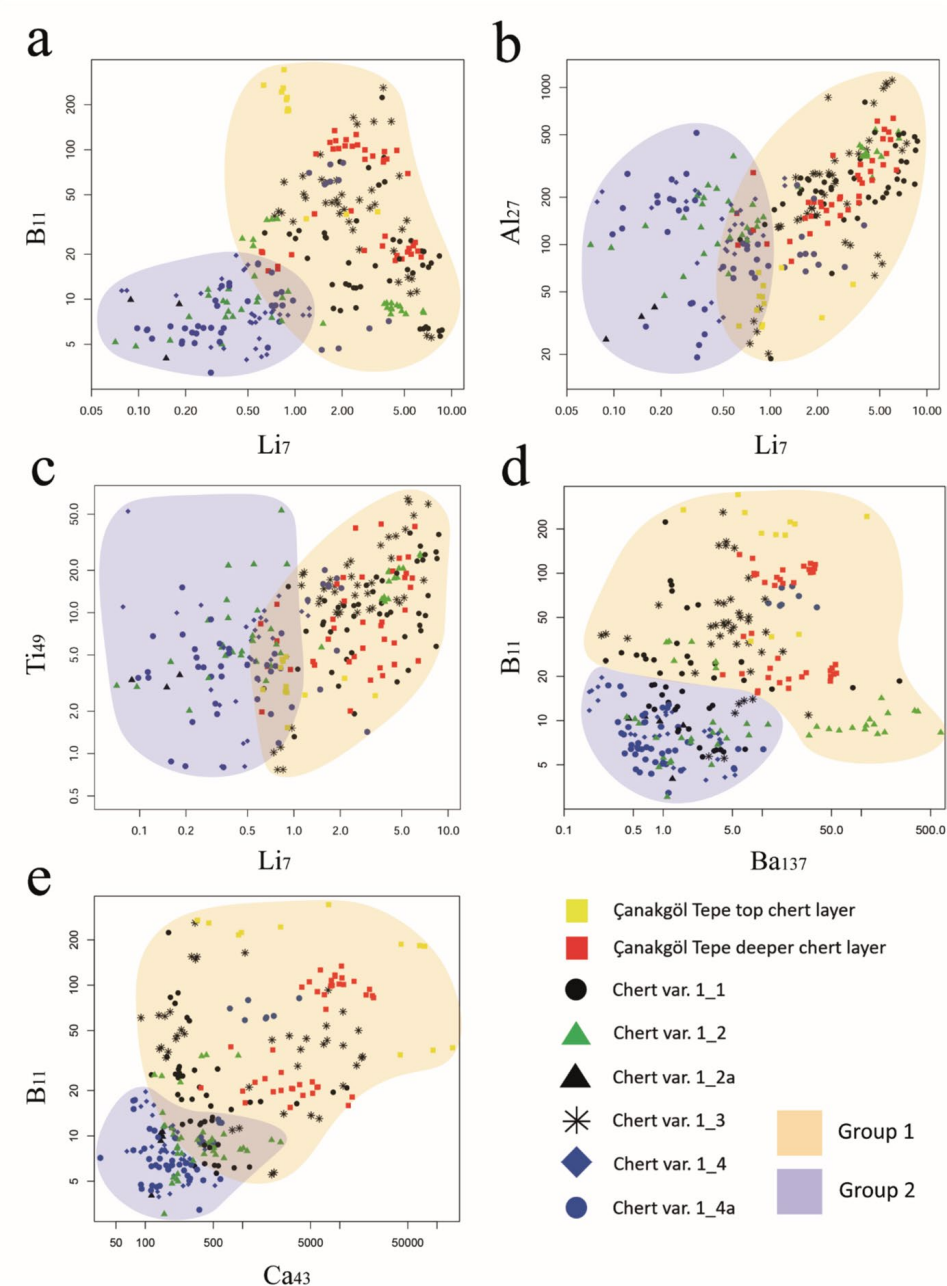
Trace element concentration plots of the geological samples and the tested artifacts from Çukuriçi Höyük. **a** Lithium versus boron (Li–B); **b** lithium versus aluminum (Li–Al); **c** lithium versus titanium (Li–Ti); **d** barium versus boron (Ba–B); **e** calcium versus boron (Ca–B). Graphic: M. Brandl ÖAI/ÖAW

The distribution of lithium versus boron (Fig. 6a), aluminum (Fig. 6b), and titanium (Fig. 6c), as well as boron versus barium (Fig. 6d) and calcium (Fig. 6e), in both geological and archaeological cherts analyzed for this study reveals the rough separation of two primary data clusters, suggesting the existence of at least two distinct chemofacies from which the lithic raw materials were procured. Group 1 is defined by geological data from Çanakgöl Tepe, which are subdivided into samples from the top-chert layer and from the deeper chert horizon. The archaeological varieties 1_1 and 1_3 as well as some samples of chert types 1_2 and 1_4a display the closest chemical similarity with this data field. The second group is only represented by artifacts and includes chert varieties 1_2, 1_2a 1_4, and 1_4a.

A plausible separation between the two groups is achieved when applying a threshold for lithium with ca. 1 mg/kg and boron with ca. 20 mg/kg to determine a marine versus lacustrine depositional environment. This is in line with data acquired during our pilot studies that showed a difference between the Li and B contents by one order of magnitude (Brandl *et al.,* 2011, 60–61). Barium concentrations of over 20 mg/kg are also unlikely under freshwater conditions and indicate the presence of seawater. Consequently, the high levels of Li, B, and Ba suggest the formation of group 1 cherts in a marine environment, while data for group 2 indicate conditions better corresponding to lacustrine facies. However, samples within group 1 show significant variation in their trace element composition. Additionally, all investigated chert types contain bioclastic fragments associated with lacustrine conditions, which is inconsistent with a purely marine origin of the samples within group 1.

The high elemental concentrations of seawater proxies in group 1 can be best explained by the effects of marine transgression into a freshwater environment. This situation corresponds with observations from Sümer *et al.* (2013) and Tuncer and Tunoğlu (2014), who describe the depositional regime within this Neogene basin system as an estuary with saline input. Consequently, this is a lacustrine environment with periodical influx from salt water, and the variations in the measured trace element concentrations represent fluctuations in the seawater level for Çanakgöl Tepe and all group 1 chert samples.

#### PCA and LDA

To substantiate the geochemical findings indicated by bivariate plots using elemental couples, we performed robust principal component analysis (PCA) and linear discriminant analysis (LDA). Since the data are compositional, an isometric log ratio transformation has been applied prior to PCA (Filzmoser *et al.,* 2018).

**Fig. 7:**
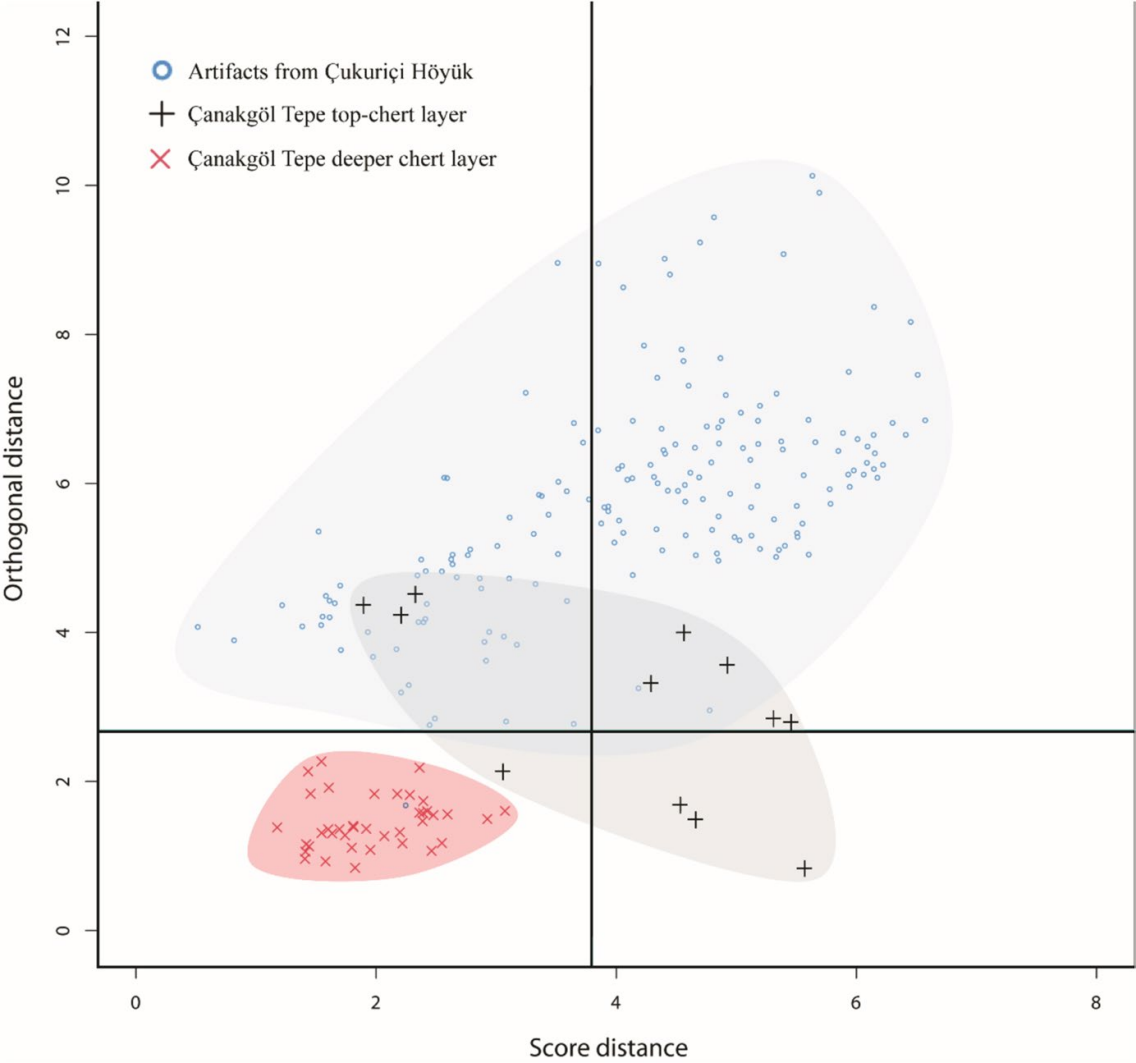
Results of robust principal component analysis (PCA) of the geological data and archaeological samples. Graphic: P. Filzmoser TU Vienna/M. Brandl ÖAI/ÖAW

Initially, the geological data set from the known geological source location at Çanakgöl Tepe (CT) was subjected to PCA. In this model, data from the geological top-chert layer and those from the chert horizon positioned deeper in the limestone sequence were displayed as two groups of training data. Subsequently, the archaeological samples were projected into the robust PCA space, and the results are presented in Fig. 7 in terms of orthogonal and score distance (Hubert & Rousseeuw, 2005).

As seen from Fig. 7, data from the top-chert samples scatter significantly, whereas those from the deeper chert horizon form a more consistent cluster. This can be explained by the fact that the deeper chert horizon was systematically sampled, whereas the top-chert layer is represented by the scarce remains of prehistoric quarrying, which left only small traces of high-quality chert distributed over a larger area on top of the hill. However, when contrasted with the archaeological artifacts, it becomes apparent that those plotting into the CT data field correspond best with the top-chert samples and that the deeper horizon does not match with any of the archaeological material. Therefore, we are able to conclude that (1) some artifacts originate from Çanakgöl Tepe and (2) it is possible to achieve geochemical differentiation between individual chert layers within a larger lacustrine depositional sequence.

In the subsequent step, we tested which archaeological samples from the identified microscopic varieties correspond to Çanakgöl Tepe and which are placed outside of the CT chemofacies, indicating an origin from a source area outside of the immediate supply zone of Çukuriçi Höyük. For this task, we applied linear discriminant analysis (LDA) to isolate two groups from the PCA results. Group 1 includes artifacts displaying chemical proximity to the geological (CT) data field, and artifacts from group 2 are defined by their largest statistical score distance from CT (Table 5). According to this prediction, only artifacts of the raw material types 1_1, 1_2, and 1_3 fall into the CT source cluster, which is in line with our preliminary hypothesis proposed in Schwall *et al.* (2020, 8–9).

**Table 5:**
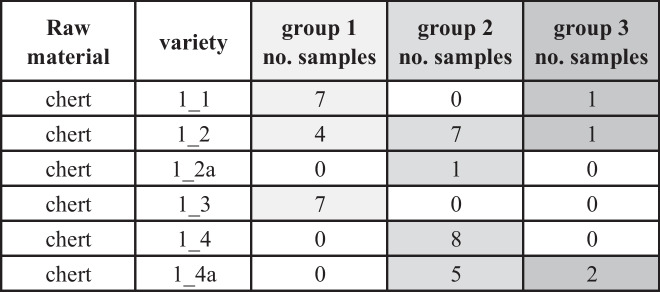
LDA assignment of the archaeological samples based on geochemical data and microfacies information. Light grey boxes indicate the samples belonging to group 1, medium grey boxes indicate group 2, and dark grey boxes indicate group 3 samples. Graphic: M. Brandl ÖAI/ÖAW

Consequently, we can identify types 1_1 and parts of 1_2 and 1_3 as raw materials of local origin; *i.e.*, they were acquired from the immediate supply zone of the archaeological site. Conversely, all other cherts are of nonlocal provenance.

### Technology

Throughout the Neolithic occupation at Çukuriçi Höyük, chert and obsidian display clear differences in their individual operational chains, target production, and use history (Milić, 2018, 2019a, 488–491; Milić, 2019b, 217–222). In contrast to obsidian, which was predominantly used for regular blade production applying pressure flaking from the very beginning at Çukuriçi Höyük and quickly surpassed all other raw materials from Early Neolithic phase XII on, the chert industry shows a more diverse development.

During the Early Neolithic phases XIII and XII, chert was used for both flake and blade production. Direct and pressure flaking were employed, with the latter prevailing in making regular blades already during the pioneering settlement phase XIII. Blanks of flakes as well as blades were modified and used, potentially reflecting particular technological needs for composite tools, some geometric microliths (lunates) and rarely projectile points. The overall proportion of retouched tools during these early phases at Çukuriçi Höyük is generally low.

Chert technology changed during the Late Neolithic settlements (phases XI–VIII), turning towards a more flake-oriented production, with a minor component of blade manufacturing predominantly through pressure flaking, less frequently direct and indirect percussion. Modified tools on flakes and blades became more frequent than during the early stages, and generally, a higher typological variability including retouched blades, scrapers, drills, and pointed tools emerged, which was likely affected also by the general number of artefacts in these phases. Altogether, up to 30% of all chert artifacts in the Late Neolithic phases were used and/or retouched (Milić, 2018, 2019a).

When applying transformation analysis (TS) to the individual chert types according to each settlement phase, particular patterns become visible. It is also apparent that phase XI does not allow for conclusive assessments for many of the chert varieties due to the small number of lithics (Table 6). As indicated in Table 6, the initial stages of the core reduction sequence are entirely missing from all chert varieties, which is in line with earlier observations and appears to be a typical phenomenon for circum-Aegean Neolithic chipped stone industries (*e.g.*, Kakavakis, 2015, 38 for Early Neolithic Greece).

**Table 6:**
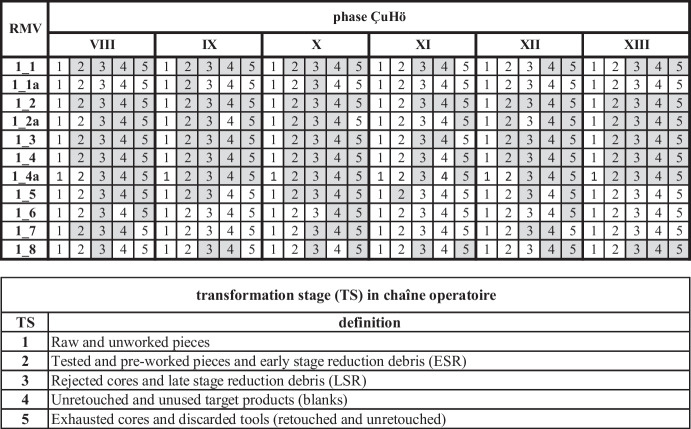
Elements (transformation stages, TS) of the chaîne opératoire present for each chert variety within each settlement phase. The elements that are present are indicated by grey boxes. Graphic: M. Brandl ÖAI/ÖAW

### Procurement

Combining the results of raw material quality, provenance, and TS, it is possible to propose the most likely procurement mode for each individual raw material type in the assemblage from a diachronic perspective (Table 7). Generally, we observe deliberate choices for each of the chert types, since the majority except variety 1_3 is of medium to high quality. The lack of initial core reduction debris for all chert varieties is an additional indication for the selective character of the overall chert assemblage since in line with Assumption #6 it is more likely that raw materials that were consistently reduced and pre-formed into cores off-site reflect the activities of skilled knappers engaged in targeted procurement.

**Table 7:**
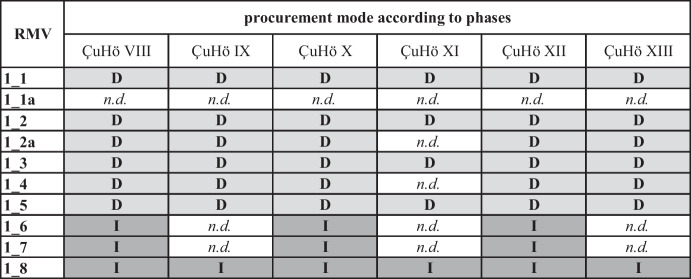
Combination of the results from quality assessment, provenance analyses, and transformation analysis of each chert variety from a diachronic perspective. Light grey boxes indicate directly procured chert types, dark grey boxes illustrate indirectly acquired varieties. Graphic: M. Brandl ÖAI/ÖAW

Code: *D*, Direct targeted; *I*, Indirect procurement; *n.d.,* Indeterminable (number of lithics too low for secure assessments)

Additionally, the geological setting with only one chert source available within the immediate supply zone of the site, which could only be reached by boat, makes random embedded procurement unlikely for the chert varieties originating from Çanakgöl Tepe. Pre-shaping of the cores would have also facilitated the transport on boats by reducing the weight and volume of the load.

As shown in Table 7, chert types 1_1 to 1_5, with the exception of 1_1a, for which we do not have sufficient data for conclusive assessments, were in all likelihood acquired through targeted procurement directly from the source(s). This is also true for variety 1_3, which is of lower quality than the other types; however, it represents the major lithic raw material still available currently in the nearby Çanakgöl Tepe chert deposit. For the chert varieties not corresponding to material from Çanakgöl Tepe (*i.e.*, 1_4, 1_4a, and 1_5), we have to take the possibility of embedded procurement into account; however, they display the same transformative patterns as the materials from the identified source. Therefore, we favor an interpretation as a direct targeted procurement scenario also for these chert types.

In contrast, the patterns emerging from the combination of highly selected elements of the chaîne opératoire and low numbers of lithics despite the high material quality suggest indirect procurement modes through external networks for chert varieties 1_6, 1_7, and 1_8.

## Discussion

This case study used the chipped stone assemblage from the coastal site of Çukuriçi Höyük to investigate chert raw material procurement as an essential part of the Neolithic economy. For this task, a fine-grained analytical protocol was employed to test raw material economic behavior from a diachronic perspective. The main questions of this study concerned the characterization and provenance of the different chert types in the assemblage, the potential procurement modes of each identified type, and the interpretation of the emerging patterns as part of the larger socioeconomic framework of this Neolithic community.

Essentially, our study produced three main results. The first and most fundamental concerns the provenance of the different chert types identified in the course of stereomicroscopic microfacies analyses. In a previous study, Schwall *et al.* (2020, 8–9) provided preliminary assessments regarding the potential sources of specific raw materials from Çukuriçi Höyük’s lithic assemblage. These initial results indicated that chert varieties 1_1 and 1_3 most likely belonged to the chemofacies defined for Çanakgöl Tepe, whereas chert types 1_2a, 1_4, and 1_4a belonged to a chemically separated source area. Chert variety 1_2 formed two microfacially indistinguishable groups, one corresponding to the Çanakgöl Tepe cluster and the other to the chemically different source area.

These observations, however, were solely based on bivariate scatter plots relying on the concentrations of trace element couples, which only allowed for very preliminary assessments and needed to be refined through in-depth statistical data evaluation. To test the working hypotheses formulated by Schwall *et al.* (2020) regarding the potential provenance of some chert raw materials from Çukuriçi Höyük and reach reliable provenance information, a series of more advanced statistical data treatments was employed for the current study. In addition to bivariate scatter plots, robust principal component analysis (PCA) was coupled with linear discriminant analysis (LDA) to achieve optimal group assignment indicative of the provenance of particular chert types. This combined statistical procedure allowed us to isolate two distinct groups from the PCA results, one of which could be identified as the local chemofacies identified for Çanakgöl Tepe, and the other containing cherts of exogenous origin. Through this sequential testing protocol, we were able to confirm our hypothesis proposed in Schwall *et al.* (2020); however, it is now based on solid statistical validation.

We were also able to demonstrate that distinct chert horizons in a lacustrine limestone sequence can be differentiated, which has not been previously attempted. This result might be of relevance for micro- and chemofacies investigations of similar lacustrine geological settings outside of the study region.

The second finding of our study is related to raw material procurement. Combining raw material quality, provenance and technological information by defining transformation stages (TS) of each individual chert variety within the assemblage in relation to knapping properties and local versus exogenous origin allows us to draw conclusions regarding the mode of procurement. This analytical procedure requires grouping all core reduction elements within the chaîne opératoire into distinct transformation stages, with the goal of visualizing patterns indicative of particular procurement strategies, which reflect deliberate choices people made. This idea is not entirely new (compare, *e.g.*, Allard & Denis, 2022); however, we achieve enhanced resolution by including more analytical parameters, resulting in a model that has the potential to reveal the dynamic character of resource management and consequently economic behavior.

The third outcome of this study is particularly intriguing, although its significance may not be immediately apparent. The emerging procurement patterns shown in Table 7 illustrate nothing less than the diachronic stability of strategies for chert resource management over a period of approximately 700 years, contrary to our initial expectation to detect stronger indications for change. Moreover, these patterns are observable from the pioneering horizon ÇuHö XIII onwards, which is even more surprising since it implies that this procurement system must have been established within a short time period upon arrival of the first settlers of Çukuriçi Höyük. Consequently, this fully developed and obviously successful economic strategy must have been based on yet unknown precursor systems, for which we do not have any material evidence. Nevertheless, it is one of the best indications for engagements of the later Neolithic settlers in this region, possibly with an elusive hunter-gatherer population leaving no physical traces since they may have only occasionally visited this coastal area (Horejs *et al.,* 2015, 304; Horejs, 2020, 166–167).

Although we are not able to answer all questions related to the beginnings of the chert economy at Çukuriçi Höyük at present, it is important to find plausible explanations for the considerably long stability of this economic system. It appears that the fundamental modes of chert procurement at Çukuriçi Höyük were established early on and essentially maintained until the end of the Neolithic period. This is of course not to say that there were no adjustments and adaptations. The procurement networks were continuously enlarged, and additional raw material types were included in the chert economic cycle, as demonstrated in Fig. 3; however, the core principles of raw material acquisition were retained. For the majority of tasks, a set of suitable locally available materials was obtained directly from their sources, whereas particular exogenous cherts probably had a more social function and were procured through external socioeconomic networks. This system might have been so effective that its overall structure remained intact and perhaps became part of a tradition that—among many other aspects—contributed to the formation of the community’s identity (Horejs, 2020, 169–171).

## Conclusion

In this study, we endeavored to visualize the processes underlying the lithoeconomy and particularly chert procurement from a diachronic perspective at a well-documented Neolithic site employing a fine-grained multiparameter approach. For this task, we went beyond mere classification by reconstructing all elements involved in this continuum and considering economic behavior as a sequence of actions within resource management. Additionally, we present a refined methodology and stringent protocol useful to answer questions relating to this challenging topic. This approach made patterns visible that were a priori undetectable using traditional techniques for processing lithic assemblages. More precisely, we used the combination of raw material provenance and technological information to assess potential procurement strategies in a semiquantitative model. For this task, we employed high-resolution geochemistry to detect trace elements via LA-ICP-MS and transformation stage analysis by classifying all technological elements of each individual chert variety detected within the chipped stone assemblage within the chaîne opératoire concept. In doing so, all chert artifacts could be assessed from a meta-level, which is typically not achieved by statistical approaches relying solely on the recorded data from litho-technological analysis. Through this analytical procedure, we were able to identify patterns we believe are indicative of specific economic behavior. Furthermore, when correctly applied, the proposed procedure enables us to examine economic behavior on a larger scale once further elements such as production, use, and distribution are included, and assemblages from contemporaneous sites are analyzed the same way and the results are compared.

For Çukuriçi Höyük, it was possible to identify eleven different types of lacustrine chert that constitute the second largest raw material group after obsidian within the chipped stone assemblage. Through the identification of the local chemofacies employing LA-ICP-MS and statistical data evaluation, we were also able to separate the local and the nonlocal components within the chert assemblage. Coupled with raw material quality and defining the transformation stages on-site, provenance information allowed us to assess the most likely procurement mode for each chert type. Eventually, the emerging patterns can be interpreted in light of the larger socioeconomic background of this Neolithic community.

Our results illustrate that chert procurement at Çukuriçi Höyük constituted a part of the economy in its own right reflecting socioeconomic dynamics. The reconstructed behavioral patterns can be considered to be a result of deliberate social choices demonstrating resilience inextricably coupled with various degrees of specialization. It becomes apparent that chert raw material procurement required considerable knowledge and a set of specialized skills. This skillset must have included the.

- Ability to identify geological settings containing deposits of particular lithic raw materials useful for chipped stone tools;
- Selection of suitable raw pieces for subsequent tool production. This needed at least medium-level knapping skills;
- Transport of the selected workpieces to the settlement, which in the case of Çukuriçi Höyük being a true coastal site had to include seafaring knowledge, since some of the sources (*e.g.*, Çanakgöl Tepe) were located offshore.

Taken all together, chert procurement at Çukuriçi Höyük has to be considered a specialized task, the foundations for which were already laid by the pioneers of the settlement with continuous further developments, while the core principles, *i.e.*, the particular procurement modalities, remained stable over the impressive time span of approximately 700 years covering the entire Neolithic sequence of the settlement.

## Supplementary Information

Below is the link to the electronic supplementary material.

Supplementary file1 (XLSX 89.1 KB)

## Data Availability

Geochemical data used for this study are provided in the “Supplement A” spreadsheet.

## Footnotes

1 In the course of several projects funded by the Austrian Science Fund (FWF) and the ERC project “Prehistoric Anatolia” directed by Barbara Horejs.

2 Geochemical analyses showed that before 6500 cal. BC exclusively Melian obsidian was used at Çukuriçi Höyük. Subsequently, very few obsidian artifacts from Cappadocian sources (Nenezi and Göllü Dağ) also appear in the assemblage; however, Melos is the by far dominant obsidian source (Milić 2019a, 488).
